# Evidence of Concurrent Stunting and Obesity among Children under 2 Years from Socio-Economically Disadvantaged Backgrounds in the Era of the Integrated Nutrition Programme in South Africa

**DOI:** 10.3390/ijerph191912501

**Published:** 2022-09-30

**Authors:** Perpetua Modjadji, Lucy Nomsa Masilela, Lindiwe Cele, Mmampedi Mathibe, Peter Modupi Mphekgwana

**Affiliations:** 1 Department of Public Health, School of Health Care Sciences, Sefako Makgatho Health Sciences University, 1 Molotlegi Street, Ga-Rankuwa 0208, South Africa; 2 Non-Communicable Disease Research Unit, South African Medical Research Council, Cape Town 7505, South Africa; 3 Research Administration and Development, University of Limpopo, Polokwane 0700, South Africa

**Keywords:** concurrent stunting and obesity, sociodemographic factors, child–mother pairs, mbombela, South Africa

## Abstract

In view of persistent stunting and increasing rates of obesity coexisting among children in the era of the Integrated Nutrition Programme, a cross-sectional study was conducted to determined concurrent stunting and obesity (CSO) and related factors using a random sample of child–mother pairs (*n* = 400) in Mbombela, South Africa. Sociodemographic data was collected using a validated questionnaire, and stunting (≥2SD) and obesity (>3SD) were assessed through respective length-for-age (LAZ) and body mass index (BAZ) z-scores. Using SPSS 26.0, the mean age of children was 8 (4; 11) months, and poor sociodemographic status was observed, in terms of maternal singlehood (73%), no education or attaining primary education only (21%), being unemployed (79%), living in households with a monthly income below R10,000 (≈$617), and poor sanitation (84%). The z-test for a single proportion showed a significant difference between the prevalence of CSO (41%) and non-CSO (69%). Testing for the two hypotheses using the Chi-square test showed no significant difference of CSO between boys (40%) and girls (41%), while CSO was significantly different and high among children aged 6–11 months (55%), compared to those aged 0–5 months (35%) and ≥12 months (30%). Further analysis using hierarchical logistic regression showed significant associations of CSO with employment (AOR = 0.34; 95%CI: 0.14–0.78), maternal education status (AOR = 0.39; 95%CI: 0.14–1.09) and water access (AOR = 2.47; 95%CI: 1.32; 4.63). Evidence-based and multilevel intervention programs aiming to prevent CSO and addressing stunting, while improving weight status in children with social disadvantages, are necessary.

## 1. Introduction

South Africa has recorded some improvements on child undernutrition, through introduction of the Integrated Nutritional Programme (INP) [1,2]. The INP, introduced in South Africa in 1994 [3], aims to guide health workers on health promotion, supplementary feeding children who are malnourished or at risk of becoming malnourished, rehabilitation of the malnourished individuals, in addition to the continuous monitoring and evaluation of the programme. Through this programme, infants between six and 12 months receive formula milk and infant cereal, and from one year onwards children receive enriched maize meal, lactose-free energy drinks, and ready-to-use therapeutic food according to government tenders [4]. However, researchers have documented an apparent lack of improvement shown by several growth indicators, despite the INP [3]. Stunting (i.e., chronic undernutrition) [5,6] remains persistent in South Africa [7,8,9], affecting 27–48% of children under five years of age [10,11]. On the other hand, overweight/obesity (12–46%) (i.e., over nutrition) is on the increase and is attributed to nutrition transition (i.e., shift from traditional diets to energy-dense diets) in the country [10,12,13].

In 2005, the coexistence of stunting and overweight/obesity in the same child was reported to be as high as 19% in Limpopo Province [10], while 18% was reported in 2010 in Mpumalanga Province, South Africa [14]. A recent secondary analysis (2016), extracted from the National Food Consumption Survey in South Africa (NFCS-FB-I) for Gauteng and Mpumalanga Provinces, showed that 68.4% of children were concurrently stunted and obese [13], indicating a public health concern. Simultaneous crises of over and undernutrition, with some children overweight while their peers suffer from stunting and wasting have been reported in other African countries such as Ghana (1.2%) [15] and Ethiopia [16], as well as in the Associations of Southeast Asian Nations (ASEAN), which include Brunei, Cambodia, Indonesia, Laos, Malaysia, Myanmar, the Philippines, Singapore, Thailand, and Vietnam [17]. An Indonesian study reported a 7.2% of CSO among children aged under 5 years [18]. In Mexico, the prevalence of CSO among children was estimated at 10% [19]. Therefore, double burden of malnutrition in ASEAN is happening in middle income countries such as Indonesia, Malaysia, Thailand, and the Philippines [17,18,20,21,22], as well as in high income countries such as Korea and Japan, and low income countries such as Brazil and Mexico [19,23].

Two different forms of malnutrition can coexist, indicating a double burden of malnutrition. The co-occurrence can be on an individual level, household, or on a country basis [14,24,25,26,27]. For the interest of this paper, the individual level of double burden of malnutrition often manifests as stunting or micronutrient deficiencies co-occurring with overweight or obesity [28]. Although, there is no uniformity on the indicators used to determine and define the double burden of the malnutrition phenomenon, particularly at an individual level [29], the literature documents a combination of height/length for age-z score (HAZ/LAZ) and micronutrient deficiency [30], BMI for age-z-score (BAZ) and HAZ/LAZ [15], weight for height z-score (WHZ) and HAZ/LAZ [28,31], and weight for age z-score (WAZ) and HAZ/LAZ [32]. The coexistence of stunting and overweight predispose to the mortality and morbidity risks, as well as poor health and cognitive development in early life. The consequences of stunting coexisting with overweight/obesity are limiting educational, social, and economic achievements of individuals across their life span. These is accompanied by an increase in the risk non-communicable diseases (NCDs) due to a high metabolic load on a depleted capacity for homoeostasis, later in adulthood [33,34,35].

New evidence points towards the shared socioeconomic, environmental, and biological factors contributing to the risk or prevalence of both stunting and overweight/obesity, although their risk factors appear distinct [24,36]. Socioeconomic status represents the underlying basic cause of malnutrition and is composed of multiple variables, such as type of sanitation facilities, source of drinking water, and housing infrastructure [37,38]. Poor socioeconomic status is directly related to illiteracy, unemployment, reduced purchasing power, and also to poor health and nutritional outcomes [39,40]. Several low-and-middle-income countries (LMICs) are experiencing socio-economic, nutritional, and demographic transitions due to economic development in the last few decades [41,42], with higher risks of coexistence of stunting with overweight/obesity reported among children [19,43,44]. Two decades after democracy, South Africa is still battling with other factors, such as poverty, inequality, unemployment, and hunger [45], while undergoing nutrition transition driving the coexistence of under and over nutrition in different settings, including rural areas [12,25,26,46]. Worth noting are the effects of the coronavirus disease 2019 (COVID-19) pandemic, which has led to high rates of unemployment and reduced food availability in the country, and ultimately reduced access to food and resultant increases in hunger among the poor [47].

Although improvement of child undernutrition, especially wasting (2.5%) and underweight (5.9%), have been recorded in South Africa, stunting remains the most persistent form of malnutrition, affecting 27−55% of children [8,9,10,11], while combined overweight/obesity has been estimated at 16.1% [48]. This is indicative that various patterns of malnutrition burden exist at the individual-level within countries or regions, changing with social development and environmental improvement in populations [49]. The most crucial goal of the Global Nutrition Targets (GNT) for 2025 is reduction of stunting and ensuring no increase in childhood overweight [50]. This is in addition to eradicating all forms of malnutrition, an essential target of the United Nations (UN) Decade of Action on Nutrition 2016–2025 and the Agenda for Sustainable Development (SDG) by 2030 [49]. Understanding the prevalence and patterns of stunting occurring alongside overweight/obesity in children is imperative for public health policy in South Africa [14]. Therefore, this study aimed to determined concurrent stunting and obesity (CSO) and related factors among children under 2 years in Mbombela, South Africa.

## 2. Materials and Methods

### 2.1. Study Design

A cross-sectional study was conducted among children aged under two years attending CHCs with their mothers in at Mbombela, in Mpumalanga Province, South Africa. This paper is part of a larger study, which determined the maternal feeding practices and nutritional knowledge, and the nutritional status of infants attending CHCs of Mbombela. The larger study was conceptualized using combinations of the UNICEF conceptual framework for malnutrition of children (immediate and underlying causes of child malnutrition) [51], WHO conceptual framework on Childhood Stunting (context on the community and societal factors, household and family factors-related causes, and consequences with an emphasis on complementary feeding) [52], and the Bronfenbrenner’s social ecological model for child growth and development (different contexts in which human development takes place, especially the influence of the primary caregiver in the early stages of a child’s life) [53,54]. The current paper reports on the prevalence of CSO and related factors using child–mother pairs. The study was conducted from May 2021 to November 2021.

### 2.2. Setting and Population

Mbombela is one of the four local municipalities in the Ehlanzeni District, situated in Mpumalanga Province of South Africa. The local municipality is situated in the north-eastern part of South Africa within the low veld sub region of the Mpumalanga Province and is the capital city of the province, which includes urban and rural population [55]. Mbombela municipality has two district hospitals and 31 primary healthcare facilities, of which six of them are where the study was conducted. The six primary health care facilities selected were those found in the deep rural areas of Mbombela, as reported by Drigo et al. [56]. The study population was children under two years attending childcare services with their mothers at the selected CHCs. The study excluded mothers who were not mentally fit to be interviewed, and those who were below 18 years and could not obtain consent from their parents/guardians to participate in the study. We also excluded children who were above 2 years, and whose biological mothers were not available to participate.

### 2.3. Sample Size and Sampling Procedure

The Raosoft sample size calculator [57] was used to calculate a sample size, considering an estimated population of approximately 3000 children attending CHCs for childcare services [58]. Within the selected six CHCs, a systematic random sampling was used to select children and their mothers. Every 3rd mother was recruited while on the queue waiting to consult, given a slip, and asked to come to the data collection site in the facility, after having been attended to, for further activities. Initially, 426 mothers with their children were recruited, and data were collected from 405 child–mother pairs. However, a final sample size of 400 was obtained, following exclusion of five questionnaires, which had over 10% of missing data, mostly including the dependent variable (i.e., LAZ and/or BAZ). The response rate of the study was 95%.

### 2.4. Data Collection and Tools

#### 2.4.1. Socio-Demographic Variables

Maternal sociodemographic data (i.e., age, marital status, education, employment, as well as household information) obstetric history (i.e., parity and obstetric complications), and child (i.e., birth) information (i.e., gender, birth weight, birth order, and delivery details) were collected using a researcher-administered, and structured questionnaire adapted from the literature [8,9,13,59] and validated through construct, content, and face validity, as well as translation and a pilot study. The questionnaire was first prepared in English and translated into the local language; SiSwati [60]. Independent translators who speak SiSwati as their mother tongue and are conversant with English did forward and backward translations of the questionnaire. An expert committee approved the final version of the translated questionnaire. The research assistants who speak SiSwati were trained on conducting the interviews in a local language before a pilot study commenced. During the pilot study, a questionnaire was pre-tested, and the research assistants were assessed while administering a questionnaire to participants in SiSwati and measuring anthropometry. Internal consistency (reliability) of a questionnaire was measured using Cronbach’s Alpha and yielded a reliability coefficient of 0.82. The feasibility of the study was tested among 30 child–mother pairs where their results did not form part of the main study. After pretesting the questionnaire, we considered minimal clarity of wording, and further simplified the layout and style of the questionnaire.

#### 2.4.2. Anthropometric Variables

Trained research assistants measured the anthropometry of children according to WHO procedures [61]. Weight was measured using a Seca 354 baby electronic scale distributed by medicare hospital equipment in South Africa, manufactured in Germany. Recumbent length (L) was measured using a Seca 210 measuring mat distributed by medicare hospital equipment in South Africa, manufactured in Germany. Results were recorded in the Section A of the questionnaire, and anthropometric data was captured on the WHO Anthro software v3.22 and analyzed according to WHO Z-scores classification for length-for-age (LAZ) and BMI-for-age (BAZ) [62]. The WHO defines stunting by LAZ/HAZ < −2 SD, overweight by BAZ > 2 SD, and obesity by BAZ > 3 SD [61].

### 2.5. Data Analysis

SPSS version 26.0 (IBM SPSS Statistics, Armonk, NY, USA) was used to compute descriptive and inferential statistics. A complete case analysis was used to identify participants with missing data. Questionnaires with more than 10% of missing/incomplete data, including missing information for the dependent variables, were excluded from the study (i.e., five questionnaires). Descriptive statistics (frequency, percentages, and cross tabulation) for the children’s age, and anthropometric measurements and indices were computed [i.e., medians (Interquartile range (IQR)], after data distribution was checked with a Shapiro–Wilk test. Mann–Whitney U test was used to compare medians (IQR) between two groups by sex categories, while Kruskal–Wallis test was used to compare the medians (IQR) for LAZ and BAZ by age categories. The z-test for a single proportion was applied to determine the significant difference between the prevalence of CSO and non-CSO within a population. Chi square test was used to test for the two hypotheses by comparing the percentages of children with stunting, overweight/obesity, and CSO by sex and age categories. The Pearson correlation coefficient (r) was used to determine a linear correlation between stunting, and overweight, obesity, and combined overweight/obesity. Hierarchical logistic regression analysis was used to determine the association between the CSO and the covariates. Variables that had a *p*-value ≤ 0.2 were used in multivariate logistic regression. A stepwise backward elimination procedure was employed controlling for confounding. Adjusted odds ratios (AOR) with a 95% confidence interval (CI) were generated and used to determine the independent strength of the associations, and significance was considered at *p* < 0.05.

## 3. Results

### 3.1. Socio-Demographic Characteristics of Mothers

The study consisted of children aged under two years participating with their mothers [i.e., child–mother pairs (*n* = 400)]. One hundred and eighty children [*n* = 180 (45%)] were boys and 220 (55%) were girls. The mean age of children was 8 (4; 11) months. Children were further divided into three age categories, which are 0–5 months [*n* = 155 (39%)], followed by those aged 6–11 months [*n* = 153 (38%)] and those aged 12–23 months [*n* = 92 (23%)]. Few of the children had low birth weight [*n* = 79 (20%)] and were born prematurely [*n* = 79 (20%)], while almost half (49%) were middle born babies and over two thirds were born at the clinics and hospitals (Table 1).

The mean age of mothers was 29 (25; 33) years with 250 (63%) mothers aged below 30 years and 150 (37%) aged ≥ 30 years. Almost two thirds of mothers (73%) were single, 21% had no education or reached primary education only, and 83% were unemployed. Most mothers were living in larger household (≥5 members; 76%) headed by themselves (35%) or their parents (46%). Seventy percent (70%) of mothers depended on child social grant and lived in households (57%) with a monthly income of less than R10,000.00 ($617). Thirty percent (30%) of mothers reported obstetric complications such as miscarriage and stillbirth (Table 2).

### 3.2. Prevalence of Stunting and/or Obesity

Table 3 shows the comparison of medians and prevalence of stunting and obesity among children by sex. Mann–Whitney U test was used to compare medians (IQR) between two groups by sex categories while a Chi-square test compared the prevalence. Overall, 51% of children were stunted while 54% obese. No significant differences were observed for medians and prevalence by sex.

Table 4 shows the comparison of medians and prevalence of stunting and obesity among children by age. Kruskal–Wallis test was used to compare the medians by age categories while a Chi-square test was used to compare the prevalence. The median of LAZ was below −2 SD for children aged 6–11 months with a significant higher prevalence of stunting (64%) compared to those aged 0–5 months (46%) and ≥12 months (35%) (*p* = 0.001). The medians of BAZ were above 2 SD for all ages with significant higher prevalence of obesity among children aged 6–11 months (63%) and ≥12 months (68%).

**Figure 1:**
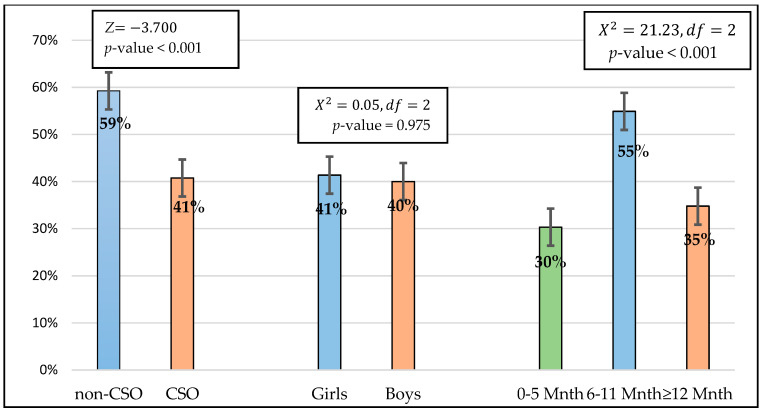
Prevalence of CSO for all children, by sex and age.

In Figure 1, the prevalence of CSO among the under two years was 41%. There was a significant difference between the prevalence of CSO and non-CSO (*p*-value < 0.001) using a z-test for a single proportion. Testing for two hypotheses using the Chi-square test, it was found that there is no significant difference of CSO between boys between boys (40%) and girls (41%). Again, there was a significant difference of CSO (*p*-value < 0.001) by age with more children aged 6–11 months having the highest prevalence of CSO (55%) compared to those aged 0–5 months and ≥12 months (*p* < 0.005).

### 3.3. The Relationship of Stunting, Overweight, and Obesity, and CSO with Covariates

The Pearson correlation coefficient (r) was used to determine a linear correlation between stunting and overweight, obesity and combined overweight/obesity among children. Results showed that stunting was significantly associated with overweight (r = 0.244, *p* < 0.001) and strongly with combined overweight/obesity (r = 0.445, *p* < 0.001) and obesity (r = 0.576, *p* = 0.036) among children (Table 5).

In the final logistic regression model, factors associated with CSO were employment status and educational level. The low risk of CSO was observed for children whose mothers were employed in both unadjusted and adjusted models (OR = 0.35; 95%CI 0.16–0.80, AOR = 0.34; 95%CI 0.14–0.78). The lower odds for CSO was observed for children whose mother completed primary school as compared to those with no education for both unadjusted and adjusted models. For the unadjusted and adjusted model, access to water was significantly associated with CSO for all children (Table 6).

## 4. Discussion

This study determined concurrent stunting and obesity (CSO) relative to sociodemographic factors using child–mother pairs in Mbombela, South Africa. Both the prevalence of stunting and obesity among children were high, while thinness, and overweight were low. A high prevalence of stunting in the current study conforms with the prevalence reported in the previous studies in South Africa, ranging between 22% and 55% [8,9,10,11]. In addition to poor cognition and educational performance, the consequences of stunting include low adult wages and lost productivity [63]. The implications of stunting are largely irreversible and have negative consequences through to adulthood [64]. The literature has shown that childhood stunting is associated with impaired fat oxidation, a factor that predicts obesity in other at-risk populations [65].

On the other hand, over-nutrition rates in South Africa are approximately 13% among children under five years [58], attributed to the consumption of infant formula milk because protein and energy intake are higher among infants who are formula-fed [66,67]. Ong and Loos [68] suggest that the greatest risk is among children who gain weight rapidly in the first week of life. Furthermore, the risk increases by 60% if the duration of rapid weight gain is increased from one to two years. The literature documents twice to thrice odds of rapid weight gain among children for developing obesity in later life [68]. Overweight/obesity in early childhood increases the risk for adult obesity, as well as associated conditions like high cholesterol, diabetes, and high blood pressure [69]. All of these are conditions on the rise in South Africa [70]. Hence, similar to other studies [52,53], we emphasize exclusive breastfeeding for the first six months of a baby’s life to protect against overweight and obesity in childhood, in addition to numerous other positive effects.

Our study provided evidence of a high prevalence of CSO among children aged under two years, indicating the double burden malnutrition on an individual level. Mamabolo et al. [71] have suggested that the high prevalence of CSO among children (≤3 years) in the Limpopo Province of South Africa, might be due to the poor quality of the diet the children consume, usually made up of typically low in animal protein, high in carbohydrate, low in fat, and micronutrient deficient. The authors further hypothesized that the lack of animal protein inhibited linear growth, while the high carbohydrate content of the diet allowed for fat deposition and obesity [71]. Similar reports on the double malnutrition have been noted in LMICs including, South Africa and Africa countries, with variations in the prevalence attributed to the socio-economic difference across the countries [72], and also implicating the nutrition transition [14,26,73,74]. Overall, the double burden of malnutrition increases the risk of mortality, morbidity, and poor cognitive development. Poor health and development in early life can limit the educational, social, and economic achievements of individuals across their life span and increase the risk of poor adult health [33,34]. There is a possibility that the prevalence of CSO is likely to continue to rise in South African children due to changes in lifestyle and dietary patterns, affecting feeding practices. Hence, efforts must be made to understand the pathogenesis of these conditions if we are to develop appropriate preventive strategies.

Stunting occurs simultaneous with overweight/obesity because of the accumulation of excess body fat [75], especially among the girls [76]. Further possible mechanisms entail the occurrence of the abdominal obesity originating from infant undernutrition, which promotes changes that are long-term and generated by metabolic adaptations [77]. Other reports have indicated that the reprogrammed hypothalamus caused by early malnutrition during pregnancy, could lead to obesity resulting from an altered appetite control [78]. Additionally, the mechanisms include a combination of poor dietary intake and delayed growth and an altered hormonal response. Children with stunting have reduced lean body mass, which causes the basal metabolic rate and physical activity to decline. Furthermore, linear growth potential is different with the deposition of adipose tissue when the energy intake is adequate [79,80]. This is caused by poor essential nutrients required for linear growth, rather than an increase in the adipose tissue. Furthermore, early nutritional programming may limit linear growth through hormonal effects, yet without a possibility of weight gain. Stunting is previously known to be related to access to food, although the connection might not be evident, especially in countries experiencing the nutrition transition [27]. It is clear thus far, that the determinants of both stunting and obesity might overlap, both predisposing to unfavorable outcomes.

Most mothers in the current study were from poor socio-economic backgrounds, characterized by high rates of singlehood, with no education or reached primary education only, being unemployed, households with a low monthly income, yet larger household size, dependence on child social grants, and lack of spouses as household heads suggesting no spousal support. The possibility is that this kind of a circumstance makes households vulnerable to food insecurity. South Africa is food-insecure at household level, due to socio-economic factors that limit access to food to a large number of people who live below the poverty line [81]. In the context of food insecurity when adequacy and accessibility of food are impaired, the mother’s decision-making ability for effective infant feeding is also disrupted [82]. Studies in South Africa have associated stunting with poor feeding practices in terms of delayed introduction of solid foods, and inappropriate food supplementation during the weaning period when infants should undergo a transition from exclusive breastfeeding to including complementary foods in their diet [8,71,83,84]. It seems as if social disadvantages expose households to poor feeding practices, especially when complementary feeding is inadequate and introduced inappropriately, ultimately leading to a child not meeting nutritional demands.

Our data showed associations of CSO by gender and age, with employment, education, and water access. There are few reports on the risk factors for CSO during childhood [18]. The variability in economic status in the current study is similar to statuses in other populations, such as the Mexican Americans and black Americans with regard to low family income, which might affect food sufficiency [85]. The significant association between perceived social status and increased risk for CSO among children suggests that even within a very poor population, there may be additional risk for families living at the lower end of the socio-economic spectrum. This trend has been observed in the United States where children in low-income families are more likely to be overweight than children in families with a high income [85]. Like in other developing countries, such as Indonesia [18], the education status of a parent is an important issue in South Africa. Our study has revealed that children whose parents had no school education were faced with CSO compared to other education levels, in line with the study conducted among Ethiopian [86], and Myanmar and Pakistani households [87]. Therefore, increase maternal education might be a significant factor that decrease the risk of CSO.

In a South Africa nutrition study, Mushapa et al. [88] has alluded the fact that a low literacy rate among women can contribute to caregivers getting jobs with very low minimal wages, which might result in low income when compared with their counterparts. Higher levels of maternal education were also observed in other studies to be protective against CSO in Mexico [19], Bangladesh and Indonesia [89], and China [44]. Further research concluded on the fact that primary school is not sufficient enough for protecting against the double burden of malnutrition among child-mother pairs, child pair, but rather that specific child feeding practice and nutrition related education have to be incorporated as a strategy to reduce the burden [86]. We emphasize educating mothers as one of the keyways through which every nutrition related policy, programs, and intervention can make a change.

## 5. Limitations

In terms of limitations, the study may not necessarily represent the entire population of children aged under two years in Mbombela, since only children attending CHCs located in deep rural areas participated in the study, hence, there are constraints on generalizability. A non-probability selection of facilities based on setting is mutually exclusive and might have introduced bias. As a result, inclusion of facilities from different settings would offer a better representativeness and a precise degree of confidence required for making an inference. However, conducting research in a rural setting is an accepted criteria supported by conceptual theoretical frameworks. The literature documents malnutrition more in the poor socioeconomic status environments typical at rural localities than in the urban. Although a systematic random sampling technique was used, mothers who attended the facilities used in this study should have an equal chance of being chosen, and thus a larger sample size so as not to skew the results is necessary. Causality could not be made from the results due to the cross-section of the study, hence the study could only report on inferences. However, application of further random sampling when selecting mothers minimized selection bias. Relying exclusively on mothers’ self-reports might be affected by social desirability, and introduce recall and reporting bias, even with assurance of anonymity and confidentiality. The conclusion of this study provides evidence on the current existence of CSO, despite South Africa continuously attempting to address all forms of malnutrition among children through the Integrated Nutrition Programme.

## 6. Conclusions

Our study highlights the re-emerging and persistent issue of the double burden of malnutrition (i.e., stunting and obesity) in South African children from poor socioeconomic backgrounds. The interventions that aim to correct under nutrition in early life need to emphasize the importance of both linear growth and appropriate weight. Possibly the parental education, especially of mothers, regarding the importance of healthy feeding practices, is important. Although to some extent, fathers provide household decisions, and contribute more income [90], while mothers spend considerably more time with their children than fathers do, hence, in most cases, they are held more accountable for the health, nutrition, growth, and development of their children, as alluded by other researchers [91,92,93]. Furthermore, the complex relationships between and within the levels and determinants of stunting and obesity make it difficult to resolve the onset or to reverse CSO. Therefore, evidence based and multilevel intervention programs aiming to prevent CSO and addressing stunting while improving weight status in children with social disadvantages, should be a public health priority to curb the dual problems of malnutrition. Finally, a focus on adequate prenatal care, improving breastfeeding, and complementary feeding practices should not be neglected. Prospective studies are necessary to study the economic status, considering the wealth index, in relation to the coexistence of both under and over nutrition among children from both rural and urban contexts.

## Figures and Tables

**Table 1:** Characteristics of children.

Variables	All *n* = 400	Frequency *n*	Percentage %
Sex	Boys	180	45
	Girls	220	55
Age categories (months)	0–5	155	39
	6–11	153	38
	≥12	92	23
Birth weight (grams)	<2500	79	20
	≥2500	321	80
Baby born full term	No	79	20
	Yes	321	80
Place of delivery	Hospital	200	50
	Clinic	153	38
	Home	47	12
Birth order	First	91	23
	Middle	195	49
	Last	114	28

**Table 2:** Characteristics of mothers.

Variables	All *n* = 400	*n*	%
Mothers’ Age (years)	<30	250	63
	≥30	150	37
Parity	1–2	253	63
	>2	147	37
Obstetric complications	No	282	70
	Yes	118	30
Marital status	Single	292	73
	Married	56	14
	Divorced	52	13
Education level	No education	72	18
	Primary education	50	13
	Secondary education	158	40
	Completed grade 12	58	15
	Tertiary	62	16
Employment status	Employed	83	21
	Unemployed	317	79
Household head	Self	138	35
	Spouse	53	13
	Parents	183	46
	Grandparents	16	4
	Relatives	10	3
Household members	<5	98	24
	≥5	302	76
Household income (monthly)	<R1000 ($61.70)	61	15
	R1001–R5000 ($61.76–308.40)	61	15
	R5001–R10,000 ($308.56–617.00)	109	27
	R10,001–R15,000 ($617.06–925.50)	93	23
	>R15,000 ($925.50)	76	19
Type of house	Mud/shack	77	19
	RDP	45	11
	Brick	278	70
Electricity access	No	68	17
	Yes	332	83
Water access	No	121	30
	Yes	279	70
Refrigerator use	No	77	19
	Yes	323	81
Type of toilet	Pit	338	84
	Flush	62	16

$ stands for United States dollar (s), and RDP stands for Reconstruction and Development Program.

**Table 3:** Comparison of medians and prevalence of stunting and obesity among children by sex.

Malnutrition Indicators	All *n* = 400	Boys *n*= 180	Girls *n* = 220	*p*-Value
Age (months)	8 (4; 11)	8 (4; 12)	8 (4; 10)	0.462
Weight (g)	10 (6.9; 11.3)	10 (7; 11.6)	9 (6.8; 11.2)	0.234
Height (cm)	65 (55; 70)	66 (56; 72)	64 (54; 69)	0.139
LAZ—mean	−1.36 (−4.61; 1.17)	−1.54 (−4.95; 1.37)	−1.21 (−4.44; 1.03)	0.127 0.975
Normal	111 (28)	50 (28)	61 (28)	
Stunting	202 (51)	90 (50)	112 (51)	
Tallness	87 (22)	41 (22)	47 (21)	
BAZ—mean	3.38 (1.48; 5.24)	3.38 (1.52; 4.76)	3.38 (1.46; 5.43)	0.988 0.191
Normal	61 (16)	21 (12)	20 (19)	
Thinness	16 (4)	10 (6)	6 (3)	
Overweight risk	51 (13)	22 (13)	29 (13)	
Overweight	51 (13)	27 (15)	24 (11)	
Obesity	212 (54)	95 (54)	117 (54)	

**Table 4:** Comparison of medians and prevalence of stunting and obesity among children by age.

Malnutrition Indicators	0–5 Months *n* = 155	6–11 Months *n* = 153	≥12 Months *n* = 92	*p*-Value
Weight (g)	6 (4.9; 7.2)	9 (8.4; 10.2)	15 (12.4; 18.4)	0.001 ***
Height (cm)	56 (51; 60)	64 (57; 69)	81 (68; 93)	0.001 ***
LAZ—mean	−1.51 (−3.62; 0.46)	−2.23 (−5.24; 0.17)	0.35 (−4.89; 4.18)	0.001 *** 0.001 ***
Normal	66 (43)	34 (22)	11 (12)	
Stunting	72 (46)	98 (64)	32 (35)	
Tallness	17 (11)	21 (14)	49 (53)	
BAZ—mean	2.24 (0.52; 3.77)	3.66 (2.02; 5.74)	4.79 (2.68; 6.94)	0.001 *** 0.001 ***
Normal	42 (28)	10 (6)	9 (10)	
Thinness	4 (3)	12 (8)	(0)	
Overweight risk	27 (18)	15 (10)	9 (10)	
Overweight	21 (14)	19 (13)	11 (12)	
Obesity	55 (37)	95 (63)	62 (68)	

*** *p*-value < 0.001.

**Table 5:** Association of stunting and overweight/obesity among children.

Over Nutrition Conditions	Stunting	*p*-Value	*n*
Obesity	0.576	0.001 ***	223
Overweight/obesity	0.445	0.001 ***	263
Overweight	0.244	0.036 **	74

** *p*-value < 0.05, *** *p*-value < 0.001.

**Table 6:** Association of CSO with selected co-variates among children.

Parameter	OR (95CI%)	AOR (95 CI%) for Gender	AOR (95 CI%) for Gender and Age
CSO			
Employed (yes)	0.35 (0.16; 0.80) **	0.34 (0.15; 0.81) **	0.34 (0.14; 0.78) **
Mother’s age (>29 years)	0.75 (0.48; 1.16)	0.75 (0.48; 1.16)	0.72 (0.46; 1.14)
Education level			
No education			1 [Reference]
Tertiary	0.48 (0.16; 1.42)	0.47 (0.16; 1.40)	0.44 (0.14; 1.36)
Completed grade 12	0.46 (0.17; 1.23)	0.46 (0.17; 1.21)	0.48 (0.17; 1.32)
Secondary school	0.53 (0.22; 1.30)	0.53 (0.21; 1.30)	0.58 (0.20; 1.32)
Primary school	0.38 (0.14; 1.02) *	0.38 (0.14; 1.02) *	0.39 (0.14; 1.09) *
Household Head			
Self			1 [Reference]
Grandparents/Relatives	0.53 (0.19; 1.42)	0.53 (0.19; 1.41)	0.62 (0.23; 1.70)
Parents	0.81 (0.46; 1.42)	0.81 (0.46; 1.41)	0.80 (0.45; 1.42)
Spouse	0.89 (0.37; 2.02)	0.86 (0.37; 2.01)	0.81 (0.34; 1.96)
Type of house			
Mud/shack			1 [Reference]
Brick	1.27 (0.52; 3.15)	1.27 (0.51; 3.13)	1.20 (0.47; 3.07)
RDP	0.90 (0.34; 2.38)	0.90 (0.34; 2.37)	0.87 (0.31; 2.39)
Household size (≥5)	0.65 (0.28; 1.52)	0.65 (0.28; 1.54)	0.56 (0.23; 1.36)
Number of children (≥2)	1.07 (0.49; 2.35)	1.06 (0.48; 2.33)	1.11 (0.49; 2.49)
Water access (yes)	2.28 (1.25; 4.12) **	2.33 (1.27; 4.27) **	2.47 (1.32; 4.63) **

* *p*-value < 0.1, ** *p*-value < 0.05, and RDP stands for Reconstruction and Development Program.

## Data Availability

The dataset for children and their mothers generated and analysed during the current study are available from the corresponding author upon reasonable request.
